# Authors’ Reply: Addressing the Digital Divide Among the Older Population Presents a Substantial Challenge

**DOI:** 10.2196/72565

**Published:** 2025-03-28

**Authors:** Lakshmi Kannan, Tanvi Bhatt

**Affiliations:** 1 Department of Psychology Bouve College of Health Sciences Northeastern University Boston, MA United States; 2 Department of Physical Therapy University of Illinois Chicago Chicago, IL United States

**Keywords:** exergame training, Matter of Balance, MOB, pre-frail, tele-exergame, tele-rehabilitation, gaming-based, tele-exercise, physical function, frailty, older adults, aging, physical activity, dementia, CogXergaming, telehealth, dynamic balance

We appreciate the insightful commentary on our study, “Gaming-Based Tele-Exercise Program to Improve Physical Function in Frail Older Adults: Feasibility Randomized Controlled Trial,” published in the *Journal of Medical Internet Research* [1], in the letter by Xie [2]. The feedback highlights critical issues regarding the digital divide and the role of older adults in co-designing interventions, which are pivotal for ensuring inclusive and effective digital health solutions.

We agree that addressing the digital divide remains an ongoing challenge, particularly for frail and prefrail older populations. As noted, a lack of interest in the content was a significant contributor to attrition in our study. While we ensured technological accessibility by using familiar platforms (Zoom; Zoom Communications) and step-by-step onboarding, engagement requires more than access—it necessitates meaningful content aligned with participants’ preferences and needs.

The suggestion to include older adults as co-designers in the development process is compelling and aligns with the principles of participatory design. Actively involving older adults can ensure interventions are relevant, engaging, and user-friendly. This participatory approach could facilitate a shift from a passive to an active role, empowering older adults to shape technologies that serve them. We acknowledge that future iterations of CogXergaming can benefit from such collaborative input, especially from frail individuals, to improve generalizability and user satisfaction.

Due to the nature of the commercial platforms used in our study, which were chosen for their cost-effectiveness and broad accessibility, tailoring the protocol based on participant feedback was not feasible. However, participants were provided with a choice of games to ensure some degree of personalization within the constraints of the available technology. To enhance the scalability, accessibility, and adoptability of CogXergaming interventions, future efforts should focus on developing cost-effective, customized platforms that allow for greater adaptability and responsiveness to participant preferences and needs. Such platforms could provide more tailored interventions while maintaining affordability, thus bridging gaps in engagement and accessibility.

The commentary further underscores the importance of exploring education levels and their relationship with learning curves. Although we reported participants’ education in the section on demographic characteristics, we did not conduct correlation analyses with intervention outcomes. This is an important direction for future research. Investigating the interplay between education, digital literacy, and intervention effectiveness may yield valuable insights into overcoming barriers imposed by the digital divide.

In conclusion, we appreciate the recognition of CogXergaming’s potential to bridge the digital divide among older adults. Future work will focus on integrating participatory design principles, exploring the role of education in digital adoption, and refining interventions to address engagement barriers. This collective effort will contribute to more inclusive and equitable technological solutions for older adults.
